# It Is Tough and Tiring but It Works—Children’s Experiences of Undergoing Radiotherapy

**DOI:** 10.1371/journal.pone.0153029

**Published:** 2016-04-07

**Authors:** Gunn Engvall, Charlotte Ångström-Brännström, Tara Mullaney, Kristina Nilsson, Gun Wickart-Johansson, Anna-Maja Svärd, Tufve Nyholm, Jack Lindh, Viveca Lindh

**Affiliations:** 1 Department of Women´s and Children´s Health, Uppsala University, Uppsala, Sweden; 2 Department of Public Health and Caring Sciences, Uppsala University, Uppsala, Sweden; 3 Department of Nursing, Umeå University, Umeå, Sweden; 4 Umeå Institute of Design, Umeå University, Umeå, Sweden; 5 Section of Oncology, Department of Oncology, Radiology and Clinical Immunology, Uppsala University Hospital, Uppsala, Sweden; 6 Department of Oncology, Radiumhemmet, Karolinska University Hospital, Stockholm, Sweden; 7 Department of Radiation Sciences, Umeå University, Umeå, Sweden; University of York, UNITED KINGDOM

## Abstract

Approximately 300 children ages 0 to 18 are diagnosed with cancer in Sweden every year, and 80 to 90 of them undergo radiotherapy treatment. The aim was to describe children’s experiences of preparing for and undergoing radiotherapy, and furthermore to describe children’s suggestions for improvement. Thirteen children between the ages of 5 and 15 with various cancer diagnoses were interviewed. Data was analyzed using qualitative content analysis. The findings revealed five categories: positive and negative experiences with hospital stays and practical arrangements; age-appropriate information, communication, and guidance to various degrees; struggle with emotions; use of distraction and other suitable coping strategies; and children’s suggestions for improvement during radiotherapy. An overarching theme emerged: “It is tough and tiring but it works”. Some key areas were: explanatory visits, the need for information and communication, being afraid, discomfort and suffering, the need for media distraction, dealing with emotions, and the need for support. A systematic, family-centered preparation program could possible help families prepare and individualized distraction during radiotherapy could contribute to reducing distress. Further studies with interventions could clarify successful programs.

## Introduction

Each year, approximately 300 children in Sweden are diagnosed with cancer [1], and 80 to 90 are treated with radiotherapy (RADTOX registry, unpublished). Radiotherapy can be used as a single primary therapy or in combination with chemotherapy and/or surgery [2]. The treatment and care of children with cancer is complex and can last for years. In Sweden, children are treated at six centers due to the demand for experienced medical staff and specialized equipment. Many children and their families must travel long distances for treatment, leaving a familiar environment and hospital. Being diagnosed and undergoing cancer treatment affects a child’s life in many ways–physically, psychologically and socially–and children use a range of coping strategies to face their illness and treatment [3–5]

Radiotherapy as a curative treatment involves delivering a dose of radiation that is high enough to kill the tumor while sparing as much of the healthy normal tissue as possible from the sequelae of the radiation [6]. Pediatric radiotherapy can be challenging for several reasons. During the treatment the child is separated from the parents; separation anxiety can cause immense stress for the child [7]. Children may be afraid of the radiotherapy machines, and separation from parents can heighten that fear [8, 9].

During radiotherapy the child must lie still; immobilization devices–such as a mask or vacuum bags, depending on the tumor localization–are used [6]. The requirement for positioning and patient compliance has increased as techniques have become more complex [10]. Anxiety can make it difficult or impossible for children to be left alone during treatment; as a result, sedation or anesthesia is used to put the child to sleep for the procedure [7, 11].

There are several reasons to avoid sedation and anesthesia for children undergoing radiotherapy treatment. The child can experience much distress during the procedure to be sedated or anesthetized, nutritional status can be affected, and sleep patterns can be disturbed. The daily routines for the child and family are disrupted. Furthermore, sedation and anesthesia is time-consuming and costly [7, 11, 12].

Children react individually to the radiotherapy procedure based on age, their developmental stage, individual coping skills and the environment [13, 14]. Age-appropriate information and preparation, support, encouragement, and talking to someone who has been through treatment were seen as important by children [7, 15]. Additionally, anxiety and discomfort can be decreased through play and distraction; information through play therapy, storytelling, booklets and film; listening to music; good collaboration with the staff of the radiotherapy department; and visiting the radiotherapy department [9, 10, 14]. Proper knowledge of child development, child psychology and distraction techniques can provide child-centered care and minimize harm [14]. Family involvement in the process is seen as increasing children’s well-being. Being informed and being prepared prior to radiotherapy treatment decreases anxiety both in children and parents [9, 15].

Although the radiotherapy process is an encounter between advanced technology and the child, few studies have investigated the child’s view of this procedure. Soanes et al. found that children with brain tumors undergoing radiotherapy treatment experienced discomfort in treatment and suffered from fatigue, headaches and other symptoms. Play and school rooms, toys, and organized activities helped maintain a sense of normality and provided distraction during treatment. The children suggested improvements to the environment such as waiting areas, ward décor, and games, in addition to having a parent close by, which provided the children with comfort and support, and helped them sleep at night [15].

The majority of published research in children with cancer focuses on children in a pediatric oncology setting, in particular as regards children’s and parents’ experiences of diagnosis and treatment, communication, information and palliative care. Few studies describe children’s experiences of radiotherapy. In the Swedish context there are–to our knowledge–no published descriptive studies of children’s experiences of radiotherapy. The aim of this study was to describe children’s experiences of preparing for and undergoing radiotherapy. A secondary aim was to describe children’s suggestions for improvement.

## Materials and Methods

This study, part of a larger multicenter study, is a qualitative descriptive study of children’s experiences of radiotherapy. Children from three of the six invited radiotherapy centers in Sweden accepted participation and were approached for interviews.

### The process of undergoing radiotherapy

The established routines for preparing child and family differ in some aspects among the three centers. At each center, brief general information about radiotherapy and scheduling is provided by physicians or nurses at the department of pediatric oncology. The parents and the child are given a small booklet containing general information about cancer therapy. At all centers, parents and children have the opportunity to visit the radiotherapy unit and meet the staff, as well as to take a look at the machines and treatment room a few weeks prior to the start of treatment. One center also has a “Children’s Web” with information about what happens in the radiotherapy unit. Detailed information about the child’s individual radiotherapy is provided at the initial meeting with the radio-oncologist shortly before the start of treatment alongside additional preparations such as tattoo marker, immobilization and CT for dose planning. Staff with experience in meeting children and family take care of practical information and show the technical facilities in the treatment room as well as the play area in the waiting room. Prior to fixation, small children are given the opportunity to play with a doll and a mask on the treatment bed in the combined fixation/CT room. During preparations and treatments, the child is allowed to take a favorite doll, stuffed toy or a CD player with music or story telling into the treatment room. One center also offered the opportunity to watch films on DVD. Parents always stay outside the treatment room during irradiation, but with the option of being able to observe their child on a screen and to talk with the child. In two centers, the children can stay in contact with their parents via a thin rope. General anesthesia or sedation, if needed, is administered to younger children. During the treatment period, parents and child meet the radio-oncologist and pediatric oncologist once a week to discuss physical problems and other questions about treatment or the child’s illness. Established routines at the different centers remained unchanged during this part of the study.

### Participants and setting

Eighteen families of a total of 36 included families, with a child aged between 0 and 18 years that had been diagnosed with cancer were asked to participate in an interview study. Thirteen of the children– 6 boys aged 7 to 15 years (median 12) and 7 girls aged 5 to 15 years (median 10.5)–agreed to participate in the interview. The sample was stratified in order to include both boys and girls and to represent the three pediatric oncology centers. The children were diagnosed with acute lymphatic leukemia (n = 1), different types of brain tumors (n = 8), sarcoma (n = 3) and neuroblastoma (n = 1). The children were receiving active treatment. No children receiving palliative care were asked to participate. Before the radiotherapy, seven children had undergone chemotherapy treatment and four had undergone tumor surgery. Two of the children had had both chemotherapy and tumor surgery before radiotherapy. During the radiotherapy treatment seven of the children were also on chemotherapy. One child received anesthesia and none received sedatives.

### Ethical considerations

The parents were given written and oral information about the study by a nurse on the ward. Parents gave oral and written consent to participate and consented on behalf of their child. Thereafter the children were given age-adjusted written and oral information and were asked if they would like to participate. Children under the age of 15 gave their oral assent and children older than 15 years gave written consent to participate. Before the interview took place the interviewers asked the children again for consent. The study and this procedure was approved by The Regional Ethic Review Board, Umeå Sweden (Ref no 2012–113 31 M).

### Data collection

The interviews were conducted by the main authors, both of whom are experienced pediatric nurses, from September 2012 to June 2014. Neither of the authors were involved in the children’s daily care. The interviews were conducted at the end of the children’s radiotherapy sessions at a time and place decided by the children and their parents. The interviews with the children took place at the hospitals (n = 12) or were conducted and recorded by telephone (n = 1). Initially, there was some small talk about everyday things to allow the children and the interviewers to get acquainted before the interviews took place. The children were invited to speak about their experiences of undergoing radiotherapy. The interviews were semi-structured and an interview guide was used in order to cover the aim of the study. The interview started with “Can you please tell me how things are right now?” After that the children were asked to describe their experiences of the radiotherapy. Follow-up questions were asked, for example “What do you mean?” and “Can you please tell me more?” The language was adjusted and simplified for younger children. All children decided to have their parent/s present during the interview, except for the telephone interview. All interviews were recorded and ranged from 10 to 32 minutes.

To enhance communication, the children were asked if they wanted to make a drawing [16] and were offered colored pencils and white paper. Seven children made drawings and explained what the drawing depicted.

### Analysis

Data was divided in two age groups: six younger children, aged 5 to 10 years, median 7 years (four girls and two boys); and seven older children aged 11 to 15 years, median 14 (three girls and four boys). A qualitative content analysis as described by Graneheim and Lundman was performed [17]. First, the main authors read and listened to all transcribed analysis to get a sense of the material as a whole. The interview texts were then divided into meaning units, each comprising sentences or phrases related to the aim of the study. The meaning units were condensed, coded, compared, and abstracted. The younger children’s and the older children’s interviews were analyzed separately in order to find similarities and differences between children at different ages. Some categories were found to contain statements from only the younger children and others from only the older children. Preliminary subcategories and categories were then formulated according to the similarities and differences after the main authors reflected on and discussed the texts. From these, 21 subcategories and five categories were formulated. During the analysis, an overarching theme emerged. Quotations from the transcribed text are shown in the findings.

## Results

This overarching theme, based on the content of the categories and subcategories, capture the children’s experiences of undergoing radiotherapy: It is tough and tiring but it works. The first part–“It is tough and tiring”–represents negative experiences such as lack of information and communication, fear, anxiety, discomfort and suffering. The second part–“but it works”–represents the effort needed to overcome the negative experiences, because refusing radiotherapy was impossible: an exploratory visit, getting information and communication, and using coping strategies such as media distraction, strategies to deal with emotions, and support from parents and staff. The subcategories describe in more detail the experiences of undergoing radiotherapy by children, ages 5 to 10 and 11 to 15, and their suggestions for improvement. Check marks for the subcategories with statements from both groups and either group are shown in Table 1. The subcategories and categories are presented below, with quotations from the interviews with number, Girl/Boy, and age.

**Table 1:** Theme, categories and subcategories describing children's experiences of radiotherapy.

Theme			
It is tough and tiring but it works			
**Categories**	**Subcategories**	**Younger children 5–10 years**	**Older children 11–15 years**
Positive and negative experiences with hospital stays and practical arrangements	Appreciating activities, being bored, and disliking waiting time	x	x
	Being together with, or missing siblings and peers	x	x
Age-appropriate information, communication, and guidance to various degrees	Having/lacking/missing information and communication about what is going to happen	x	x
	Having an explanatory visit and meeting with staff at the radiotherapy ward	x	x
Struggle with emotions	Being afraid and feeling anxiety	x	x
	Disliking and accepting the mask, the dot tattoo, and the machine	x	x
	Finding the right position and remaining motionless	x	x
	Disliking the sensations		x
	Suffering physical and psychological problems to various extents		x
	Appreciating small gifts	x	
Use of distraction and other suitable coping strategies	Using a suitable media distraction	x	x
	Using problem-solving activities		x
	Using strategies to deal with emotions	x	x
	Wanting parents close by before, during, and after treatment	x	
	Seeking support from parents, staff and peers		x
Children’s suggestions for improvement during radiotherapy	Desire for facilitating routines and suitable equipment	x	
	Desire for specific, individualized, and easy-to-understand information		x
	Desire to choose the kind of distraction preferred	x	x
	Desire for gifts as encouragement	x	x
	Desire for sympathetic staff and continuity	x	x
	Desire for peers to talk to		x

### Positive and negative experiences with hospital stays and practical arrangements

#### Appreciating activities, being bored, and disliking waiting time (both groups)

When younger children have to undergo radiotherapy over several weeks, they express appreciation for the play therapy and the hospital school. The children either go to their usual school or the hospital school; in either case, school is described by the younger children as an important everyday activity, and they like to meet with class mates: “School feels normal… and my friends know everything [about my illness]” (0310G8). They appreciate leaving the hospital environment to go to the park, to feed the birds or to take a walk downtown. Older children made no statements about play therapy or the hospital school. They described preferring to live outside the hospital in an apartment together with their parent(s) because it felt more like home. They reported appreciating taking part in activities that they are used to. In both groups the children describe feeling bored at the ward because there is a great deal of waiting time: “…oh, that’s the time to wait, like, a lot of it. If you’ve decided to meet some doctor, uh, and then you have to sit in the waiting room for, like, four hours” (0106G12). Sometimes they can live at home and just visit the radiotherapy department for treatment.

#### Being together with, or missing, siblings and peers (both groups)

Younger children described enjoying being together with their siblings, and that they missed them during hospitalization: “It’s quite a long way from home… you can’t see your siblings so often…” (0102B9). Children spend time at the ward and they are together with other children playing, watching a film, and enjoying each other’s company. They long to meet with their friends, and according to the children, they are looking forward to their visits. Some of the older children state that they have visits from siblings or friends. Sometimes siblings can stay at the apartment so that the family can be together. They describe missing friends and that they cannot see them so often during the period of radiotherapy: “…boring… and then you can’t see your friends as much” (0208G14).

### Age-appropriate information, communication, and guidance to various degrees

#### Having/lacking/missing information and communication about what is going to happen (both groups)

Some of the younger children remembered they had information about the radiotherapy treatment: the staff informed them by using pictures and a book. “Yeah, I was a little nervous… there was someone telling about it” (0102B9). Some children got information from their parents and some of them say they got no information:”No, there was nobody telling about it” (0315G5) and another said: “The thing was, I didn’t know that much (0209G5). Older children described getting easy-to-understand information and having good communication with the staff, which they found was necessary. A few, however, described missing information:”… [Being prepared for the sensation of light] Eh, no, not–not the first time… I didn’t have my eye on the mask, either, so I didn’t know…” (0202G14).

#### Having an explanatory visit and meeting with staff at the radiotherapy ward (both groups)

All younger children described their visit to the radiotherapy department. They looked at the room, tested equipment and the machine and met with the staff:”The people who worked there… yeah, showed me the machine and showed me the bed I was going to lie on… it looked a little strange” (0102B9). Another child described: “There was a bench… ah, and a little hose, cords hanging” (0209G5). Meeting the staff was described as important for the children in order to feel safe. The older children described the visit–seeing the room and the equipment, and talking to the staff–as clarifying because the oral information given by staff at the ward or parents is perceived as not enough, and some wanted to test things out: “Yeah, although [*hesitates*] it’s not… it’s not the same thing, you’ve really got to try it to understand” (0106G12). They also described asking questions directly to the staff at the radiotherapy unit and getting answers to their questions.

### Struggle with emotions

#### Being afraid and feeling anxiety (both groups)

Most of the younger children described being afraid and sad during the radiotherapy treatment. They found the treatment frightening:”No, I was mostly afraid” (0310G8). Most of the children were awake and one had anesthesia. Being left alone in the room during the treatment was described as a difficult experience: “We tried to do the radiation therapy when I was awake, but… it got too lonely in the room when they [*the parents*] had to go out. It felt nice to sleep” (0310G8). Another child described:”Hm… [*thinks*] I didn’t want to be there for the radiation” (0209G5). The older children expressed feeling nervous, worried, and also suspicious, as well as experiencing some fear before the first treatment. A boy described the hesitation he felt: “From the start, I refused up until the end. It was Friday, the first Friday, so I refused to go. But I was simply forced [*laughs*]” (0101B12).

#### Disliking and accepting the mask, the dot tattoo, and the machine (both groups)

The younger children’s experiences of the mask varied. Many thought constructing the mask was difficult; the plastic material was warm and wet and caused discomfort. Some children described accepting the mask during the radiotherapy treatment although it was very tight and chafed, while others describe having difficulties breathing, and the mask as being too warm to have on and hurting: “Then it was also a little tough when the mask was fixed, because it sat really hard, that mask” (0102B9). One of the children could not overcome the fear of the mask and the only way to use it was to put it on when the child was unconscious after being anesthetized. Many of the younger children commented on the machine, describing it as scary, strange and very big: “It’s big… and then it spins around and makes sounds, it rumbles” (0205G7). The duration of each treatment affected the children’s experiences. A longer session often meant more difficult experiences with the mask and a fear of the machine; a shorter session made it easier to overcome the discomfort. Most of the older children described accepting the mask and the dot tattoo. Although some described worrying about the tattoo marker or difficulties such as the mask feeling too tight and a sensation of feeling stuck: “So… I got a goddamned mask like this… uh, then I had to have radiation while in it. That was worse. A mask, but it’s… like when you’re stuck in… it’s tight at first” 0218B15). Another described the tattoo marker: “I was nervous about the tattooing but then I saw that they were barely there” (0202G14).

#### Finding the right position and remaining motionless (both groups)

Finding the right position and remaining motionless was described as difficult by many of the younger children: “That was probably the hardest… lying still… it itches [*when you’re supposed to lie still*]” (0102B9). Some of the children found it boring to remain still: “So it’s so hard to lie still with your legs” (0209G5). One of the youngest children described no problems with being motionless: “The people working said that I was lying still… that I was good” (0205G7). For some children, however, the duration of treatment did not matter; for others, the time was perceived as long. The older children stated it was not a big deal to find the right position and to lay motionless; it often worked immediately during the first treatment. They described getting used to the process gradually, or right from the start:”I think this went well the whole time” (0208G14).

#### Disliking the sensations (11–15 years)

The older children, who experienced sensations of lights, noises, or smells strongly expressed disliking the phenomenon:”Things I react to that I don’t think are all that cool, is that it smells really bad… it smells like iron. I smell it every time… and there was a blue light, and then there was that sound. It sounded like something tooting… and then it finished, that thing with the light. That’s nice” (0101B12). Some also describe that it felt weird and that it was hard to endure the sensations.

#### Suffering physical and psychological problems to various extents (11–15 years)

The older children reported various physical symptoms. Some described losing their hair, experiencing pain, feeling weak and shaky or very tired. One boy described changes to his skin and the irritated skin he got as a result of radiotherapy. Some described nausea and vomiting, whereas others described loss of appetite, and difficulties swallowing and eating food: “My throat’s really fragile… I can’t swallow… So I don’t have a big appetite and I don’t each much at all. I try. Most of it’s difficult” (0302B15). These children also expressed how things like being tired all the time and not being able to do anything were bothersome. Experiencing nausea was experienced as stressful; being unable to eat and having food aversions affected them emotionally. Receiving negative information or having changes in treatment were stated as hard or a cause of worry: “I had radiation on my back first, because I was having chemotherapy at the same time as I was having radiation first. Because I have tumors that don’t respond to treatment. When I had to start radiation on my head. Because then it felt like it hadn’t helped anything… I also think it’s tough because I have these record doses” (0218B15). Some of the older children expressed that it felt nice and was a relief when the treatment came to an end-point: “Now it feels really nice that the radiation will soon be over” (0106G12). However, one boy expressed anxiety about the future because the treatment had not been successful: “Oh, then in two weeks the MR will x-ray again and see if it’s helped anything. If it, like, hasn’t disappeared down there where it’s sitting, it can’t be irradiated any more. That’s a little scary, because, like, then you die. Nothing can be done about it, either. It feels screwed up” (0218B15).

#### Appreciating small gifts (5–10 years)

The younger children liked getting small gifts after treatment. One of the children collected small marbles, one after each treatment. Other things, like a nice pencil and a treasure chest from which they could pick a small gift was described as something to look forward to. When treatment is done they describe celebrating and they received more gifts:”Today I got three things” (0203B7); one girl described getting a dress.

### Use of distraction and other suitable coping strategies

#### Using a suitable media distraction (both groups)

The younger children described the distractions they preferred during radiotherapy: some liked listening to music, reading a story, or watching a film. Older children described choosing whether they wanted a media distraction, and if so, what kind: listening to music from a CD or on the radio. In one case, it was possible to watch a film. Mostly, the durations were short so music was the media distraction of choice. Some children also chose to have it quiet.

#### Using problem-solving activities (11–15 years)

The older children described using actions to solve problems. When they experienced sensations of smell they described holding their noses or their breath to avoid smelling it. Other solutions occurred: “To get rid of this smell… we usually put a bit of chocolate in front of my nose [*laughs*] so it smells a bit like chocolate and I get to eat it when I’m done, so that’s why I think it’s such a good idea [*laughs*]… it doesn’t help, but it’s good [*laughs*])” (0101B12). They expressed appreciation when the staff was flexible and found solutions together with the children during the process: “…once I did it twice in one day… that was because I wanted to take a day off, because I was going away [*brief pause*] on a class trip” (0302B15).

#### Using strategies to deal with emotions (both groups)

According to the younger children some of them used internal dialogue during the treatment. They talked to themselves and felt that the time went faster that way. One child described talking to herself, as she could not talk to her parents. Another child described that he found time went faster when he was thinking about something he liked: “I think about something fun, about graffiti and ice hockey” (0102B9). Some children described bringing their stuffed toys to the radiotherapy treatment. One child stated: “My stuffed toy can stay in the same room, near me… I talk with them… my stuffed toy doesn’t get scared” (0315G5), and the stuffed toy had a mask and clothes made by the staff. Staff treated the child and her stuffed toy the same way. Another child described her stuffed dog: “I take my stuffed toy with me… a big dog” (0205G7) and she talked to the dog during treatment. The older children described different strategies for enduring treatments for weeks such as accepting, just doing it, taking it one day at a time, using relaxation techniques, or using a countdown: “Like, take it one day at a time and count, now one day is gone” (0106G12). Some of the children looked forwards and had a goal or plans about how to celebrate when treatment ends.

#### Wanting parents close by before, during, and after treatment (5–10 years)

The younger children wanted their parents close by. They described their parents going with them to the radiotherapy department, keeping them company in the waiting room and helping them get ready for treatment. After that, the parents left the room: “They just have to leave me for a bit, then they’ll come” (0315G5). The children knew why their parents could not stay with them the whole time, and said it was good to know they were outside the room even if they were separated: “They [*the parents*] are entirely outside, but there is a wall between” (0209G5). Parents could stay until the child was sedated or asleep from anesthesia. Afterwards the children described it felt good to wake up and see their parents:”…an empty room and Daddy…” (0310G8).

#### Seeking support from parents, staff and peers (11–15 years)

A few of the older children described talking to parents or staff, or having meetings with a psychologist. They appreciated being accompanied by one or both parents. In addition one of the children had a peer at the ward and they could talk to each other about the treatments: “There was another boy also, who was also getting radiation to his head, which removed a brain tumor, just like me. We meet at breakfast, at lunch, and at dinner and I’ve asked him… if he also noticed this light and smells, but he noticed the light sometimes he says, but not any smells… very different” (0101B12).

### Children´s suggestions for improvement during radiotherapy

#### Desire for facilitating routines and suitable equipment (5–10 years)

The younger children experienced that preparation prior to treatment could take a long time: “Then [*at the start*] it was nearly an hour the first time” (0102B9). They suggested that the time for preparing prior to irradiation and radiation time should be shortened: “I think it’s tough that it [*the radiation*] takes so long” (0315G5). Waiting time was also described as long and boring: “I wait and wait for it to be my turn…” (0315G5). Some of the children commented on the bed, saying it should be softer and more comfortable. The children did not like the mask. They described it as hard, and that it chafed and hurt.

#### Desire for specific, individualized, and easy-to-understand information (11–15 years)

The older children talked about specific, individualized, and easy-to-understand information about radiation, and about specific words and what they mean. Some of them gave examples of children playing computer games in which radiation was something dangerous that a person could die from: “It sounds scarier than it is. You think it’s *Star Wars*… I don’t know about this… radiation” (0202G14). The children stated that they would like to have all information so as to be prepared for sensations: “If you’re getting radiation to the face, it could be good to know there will be a bad smell or… a bad taste, oh. A little of that, practical.” (0106G12). Children also stated they wanted to be prepared during the session: “Talking in the speakers, that helped a lot. Because then you’re prepared…. Otherwise it’s like a surprise, you’re lying there and suddenly it just starts the radiation” (0103B13).

#### Desire to choose the kind of distraction preferred (both groups)

The young children wanted to decide themselves what kind of distraction they wanted during the treatment. They wished to have more films, music and fairy tales to choose from when they were undergoing radiotherapy. Some children took their own films with them as distraction during the treatment. Play was mentioned as an important preparation prior to the radiotherapy session. Older children also expressed the desire to choose if and what kind of distraction that they like. Having the music they preferred was perceived as nice.

#### Desire for gifts as encouragement (5–10 years)

The younger children liked to receive small gifts as encouragement, and looked forward to post-treatment. They wanted gifts that suited both girls and boys. Older children described small presents being appreciated. Sometimes something extra was valuable as a surprise.

#### Desire for sympathetic staff and continuity (both groups)

In both groups the children emphasized continuity in the staff. Treatment was difficult to go through when they did not know the staff: “She wasn’t the one I knew” (0203B7). Furthermore, they wanted the staff to be kind and not in a hurry. One of the older boys thought it was very good to have continuity among the staff and to meet the same person each time: “I think you should continue with that” (0101B12).

#### Desire for peers to talk to (11–15 years)

Among the older children, one of the boys described it being nice to have peers to talk to who understand exactly what radiotherapy is like. He therefore suggested that all children should have that opportunity.

## Discussion

The main findings were that radiotherapy includes struggle with emotions and is perceived as “tough and tiring but it works”. Although the children described facilitating and comforting measures taken by staff and parents, there were still several elements that caused distress for the children.

Children in both groups described receiving information, communication and guidance in various ways. According to the participants, the visit to the radiotherapy ward was appreciated and provided an understanding that pictures and words couldn’t. This indicates that the staff has adjusted to the right level and had a well thought-out approach to children. As the experience from the children varied in the present study, however, additional education in child development and child psychology as described by Harvey-Lloyd [14] may be needed for staff to have the skills to reach each single child. The procedure–fixation, the mask, the machine, the treatment bed, and being alone in the room–may cause fear [8, 9] and has become complex [10]. A family-centered approach could provide guidelines on how to treat children and their families [18]. From this study, it is not possible to explain why information was missed in those cases. Sometimes it may have been lack of time, new staff in charge not knowing the child, or lack of skills.

Children expressed their struggles. They described feelings of anxiety, fear, and hesitation concerning the start of treatment. Some did not understand the word radiation and associated it negatively with computer games. Minimizing anxiety is a goal for health professionals; earlier studies have shown that being well prepared decreases anxiety in children [9, 15]. At the start of treatment, however, most children may feel nervous because of the new unknown situation they have to deal with. One child refused at first, but was persuaded to do it because radiation is necessary for achieving a cure. It seems that some of the children did not clearly understand the given information. As not to miss valuable information, a systematic pre-radiotherapy intervention program–a checklist–for making sure a child is well prepared regardless of age and developmental stage as well as the type or time required for the treatment would be of use. Trying the mask on at home could be one possibility included in the program. Pre-operative intervention, for example practicing with the anesthesia mask, reduced anxiety in children [19]. Furthermore, age-appropriate preparation reduced the need for anesthesia when preschool children underwent magnetic resonance imaging [20]. Reducing anesthesia is also cost-effective [7, 11, 12]. During radiotherapy it is extremely important to find the exact position in order not to damage tissues around the exposed area or to miss the target. Technological research into finding new solutions, for example the material of the mask or other fixations, could help the children better tolerate the procedure.

The older children reported physical and psychological concerns. It was surprising that the younger children did not mention side effects as their parents did [21]. Developmental aspects can likely be an explanation of why older children can express side effects more distinctly. There are similarities and differences between subcategories reported by younger vs. older children. One possible explanation is developmental skills among children. Coping, for instance, is dependent on the development of language and abstract thinking [22]; older children therefore have more developed coping skills. Health professionals must bear in mind that the treatment procedure and side effects are very stressful for children; they need to routinely ask children directly about physical and psychological distress. The older children´s proposals for improvement were to have specific, individualized, and understandable information about radiation and side effects prior to the start of treatment. They wanted to be prepared and to know what they could expect.

The children reported using a range of strategies during the treatment. However, their proposals were to have more films, music, and fairy tales to choose from. Some children also stated that they wanted to choose whether they wanted distraction, and if so what kind they would like. The younger children wished to get nice presents. This is an area for development: first, to improve the distraction material that it would be possible to use, and then to involve each child in the decision-making process about what kind of distraction is suitable. Furthermore, other strategies to use could be described, for example internal dialogue and relaxation. The younger children expressed separation anxiety in line with other authors’ descriptions [7, 15] and their need for parental support. They describe needing to have their parents close by, which is impossible due to the risk of exposing them to radiation. One possible suggestion is the voice of the parent reading a favorite story. String as a form of maintaining contact could be of comfort to some children. A list of distraction methods suitable for different ages, providing a spectrum to choose from, could be offered. At present the younger children receive a small gift each time, which is appreciated. Having a way of counting down could also be useful in obtaining a more visual picture of when treatments will be finished. All children need support from parents, but in different ways. Older children suggested having peers to talk to; this finding is in line with others [7, 15]. A structured way of investigating the need for peers to talk to, and giving advice on how to find peers, could be helpful for some of the older children.

Finally, the practical arrangements could be developed to facilitate the process for children and their families. Children appreciate being able to play and take part in activities, going to school, having leisure time, seeing siblings and friends, and staying in homelike surroundings. According to the children, they do not like waiting times. Every improvement in the organization that minimizes waiting times will be for the better.

Some methodological considerations should be discussed. Different ages, gender, and types of treatment were included to ensure credibility [17]. During the analysis, the main authors moved back and forth within the research process, discussing data collection, analysis, and interpretation and reflecting on the findings [17]. The use of interviews was an appropriate method for gathering data; it yielded a rich amount of data to analyze [17][23]. Some children were more open to speaking whereas other more silent, depending on age or personality. The children were encouraged to speak more about their experiences through follow-up questions. One interview was conducted by telephone with a teenager owing to the great distance. Telephone interviews can produce honest discussions and rich data, and make it easy to talk because of the anonymity [24]. The telephone interview was conducted with a 15-year-old boy and lasted 20 minutes. Lack of visual cues and body language may be a weakness, however. The interviewers listened attentively to the participants and were prepared to interrupt the interview if there were signs of fatigue or upset. As the children shared their feeling on various topics, we regard the data as trustworthy. All of the children, except for the telephone interview, had a parent or both parents close by to make them feel safe. This might have influenced their answers. Sometimes, however, the parents could help the responder remember details.

The findings contribute to our understanding of children’s experiences of radiotherapy treatment. Some strengths and limitations should be noted. We interviewed children of different ages, split into two groups. One main author analyzed the interviews of the younger children; the other author analyzed the older children’s interviews. Both authors are trained and experienced in interviewing children and analyzing data. Some statements in subcategories occurred in one group and not in the other; as we interpret it, this reveals some differences that are dependent on development. The interviews were performed at the end of the radiotherapy treatment, which could be seen as a limitation, because the children might have forgotten what happened at the beginning, and as a strength because the treatment was over or soon finished and they could reflect upon their experiences when talking about them. The findings can be transferred to other similar settings in Sweden and, possibly, transferable to a broader context. We leave this for the reader to determine.

## Conclusion

Undergoing radiotherapy for children is perceived as tough and tiring, partly depending on age, development, and the kind of treatment–as well as place and time–required. An education in child development, child psychology and the psychological preparation of children for staff in radiation wards could potentially improve skills for meeting every child in an individualized and personal fashion. A systematic family-centered preparation program could possibly help families prepare and individualized distraction during radiotherapy could contribute to reducing distress. Further studies with interventions could clarify successful programs. Additional interviews of staff involved in radiotherapy could provide valuable insight about difficulties and facilitating routines when children have to undergo radiotherapy.
